# A Libellous Letter

**Published:** 1899-02

**Authors:** 


### A LIBELLOUS LETTER.

THE following quotation is taken from the December number of the *Dental Review.* It is not often that this journal is called upon to notice the anonymous attacks appearing in other journals, but this, pretending to emanate from one of the household, should receive attention, if for no other reason than to denounce it as an infamous calumny.

> " As a looker-on, though not entitled to a vote, at the late meeting of the Dental Faculties Association in Omaha, the undersigned was impressed with the thought that surely the truly good must seek other spheres than this before perfection is attained. Here are the chiefs of dental education, assembled in council of war; they discuss the physical and mental caliber of the expected new recruit, the ignorant student, as though he were the only enemy involved in the coming scrimmage. . . .
>
> " As things are now, when a college is admitted to membership,—and almost any new ' old thing' can be so admitted,—it at once becomes the peer of the best in the general estimation of the profession and State boards. It ' complies with all the rules aforesaid' in its catalogue, and then does pretty much as it pleases, knowing that the mere official cloak of membership in the Association can cover many sins of skullduggery. And thus —' Oh, the pity of it, Iago'—the College Faculties Association is used as the responsible protector, the cat's-paw of what otherwise would be, on its own merits, a disreputable institution."

It is more than probable that the foregoing was written by one not entitled to be present at the meeting of the National Association of Dental Faculties, for it is inconceivable that any member of a faculty would stoop to lower this body in the estimation of the dental profession. It was doubtless prepared by an outsider with malice aforethought, in hopes that his baseless charges would have some effect in prejudicing those not familiar with the work of this organization, and thus destroy its valuable moral influence in elevating dental education in this country.

It is needless to say that the statement that the members spent their time in discussing " the physical and mental caliber of the expected raw recruit" is false, and no one knew this better than the author of this wretched screed.

The Association of Dental Faculties was organized for the purpose of eradicating the evils entailed by loose methods of training, and in order to accomplish this time was required to perfect certain measures. The difficulties to be surmounted in accomplishing this can only be appreciated by those intimately connected with the work. That much has been done and more yet to be accomplished requires no statement or argument. The proof of the first lies in the fact that through its labors dental education in the United States to-day occupies a position quite equal, if not superior, to that of any other portion of the world. The Association of Dental Faculties has been in existence fourteen years. At the time of its organization students were being graduated under what was known as the "five-year rule," which meant, practically, one session. The requirement of two full years was the first work of this body, and from the time this was passed to the present hour it has steadily advanced its requirements.

The insinuation of this correspondent, that colleges do not live up to the requirements, is quite in line with the tone of his letter and equally devoid of truth. The editor of this journal has had better opportunities of familiarizing himself with the dental colleges of the United States than many, even of the members of the Association, and while there may be a few secret violators of its rules, as there are dishonest members of all communities, these must be exceedingly rare, and when the attempt is made, it is almost certain of discovery, and this means a risk that few care to take. In the earlier period of this organization one college conceived the idea that it could be a law unto itself, and disregard all rules. The result was, the school was expelled at once, with a subsequent financial loss so heavy that it was forced to reorganize and humbly beg for readmission.

The Association of Dental Faculties means all it says in the resolutions adopted, and every college must live up to these or suffer the consequences. This was plainly apparent at the Omaha meeting, but the correspondent probably overlooked it, or to his mind it was not important. A prominent college was reported as not having had anatomy taught by dissection of the cadaver, and it was recommended that it be suspended. A vigorous effort was made to save the college, for it was thought it labored under special difficulties, but all that could be obtained was a respite for a few months. If the rule of the Association was not then complied with, the college was to be cast out of the fold.

If the writer of the letter in the *Dental Review* has any complaints to make, the proper plan is to bring them before the committee appointed for this purpose, with all the details of violations of rules, and he can be assured that, whether high or low, the faculty offending will be called to an accounting.

It is time that the moral sense of the editors of journals should be above publishing such scurrilous letters. There is too much of this in several of the dental periodicals, and they frequently step over the border-line of the law of libel. While associations cannot be protected always by legal statutes, they, at least, are entitled to respect, and the editor who admits an article calculated to lessen the influence of a worthy organization merits and should receive severest condemnation.

